# A Lifestyle (Dietary) Intervention Reduces Tiredness in Children with Subclinical Hypothyroidism, a Randomized Controlled Trial

**DOI:** 10.3390/ijerph17103689

**Published:** 2020-05-23

**Authors:** Ellen van der Gaag, Job van der Palen, Pim Schaap, Mirthe van Voorthuizen, Thalia Hummel

**Affiliations:** 1 Department of Pediatrics, Hospital Group Twente, 7609 PP Almelo, The Netherlands; pim_schaap@hotmail.com (P.S.); kinderartsen@zgt.nl (M.v.V.); 2 Faculty of Behavioural, Management and Social Sciences (BMS), University of Twente, 7522 NB Enschede, The Netherlands; j.a.m.vanderpalen@utwente.nl; 3 Department of Pediatrics, Medical Centre Twente, 7512 KZ Enschede, The Netherlands; t.hummel@mst.nl

**Keywords:** subclinical hypothyroidism, tiredness, fatigue, lifestyle, dietary intervention, beef, vegetables, whole dairy products

## Abstract

**Purpose**: Subclinical hypothyroidism (SH) in children and adults is a subject for discussion in terms of whether to treat it or not with respect to the short-term clinical implications and consequences of SH and in the long term. If treatment with thyroxine supplementation is not indicated, no other treatment is available. We investigated whether a lifestyle (dietary) intervention improves or normalizes SH or decreases the presence of Thyroid Stimulating Hormone (TSH) and/or tiredness. **Methods**: We randomized children aged 1–12 years with SH to the control group (standard care = no treatment) or intervention group (dietary intervention). The dietary intervention consisted of green vegetables, beef, whole milk and butter for 6 months. The rest of the diet remained unchanged. We measured TSH, FreeT4, Lipid profile, Body Mass Index (BMI) and Pediatric Quality of Life (PedQL) multidimensional fatigue scale scores. **Results**: In total, 62 children were included. After 6 months, TSH decreased in both groups without a significant difference between the groups (*p* = 0.98). PedQL fatigue scores for sleep (*p* = 0.032) and total fatigue scores (*p* = 0.039) improved significantly in the intervention group, compared to the control group. No unfavorable effects occurred in the lipid profile or BMI. **Conclusion**: The lifestyle (dietary) intervention did not normalize SH and TSH levels, but it significantly reduced tiredness. These results suggest that children’s well-being can be improved without medication.

## 1. Introduction

Subclinical hypothyroidism (SH) is a biochemical condition defined as subclinical because only the serum level of thyroid stimulating hormone (TSH) is slightly increased. Other thyroid hormones like FreeT4 (FT4) are in the normal range [1]. Subclinical hypothyroidism is present in about 2% of the children compared to about 15% in adults [2,3,4,5]. Chronic auto-immune thyroiditis is the most common cause for SH (about 50%–80%), with or without high thyroid peroxidase antibodies [6]. About 1% of the children with SH develop clinical hypothyroidism [4].

In theory, a child with subclinical hypothyroidism would not have any clinical symptoms, since only the TSH is increased and the functional hormone (FT4) is not involved. Despite the apparent lack of relation between thyroid biochemical parameters and clinical symptoms, multiple complaints related to SH have been found in daily practice and medical surveys. Tiredness, weight gain, disturbed lipid profiles, impaired growth velocity, excessive sleepiness and physical weakness are described [2,7,8]. Children and adults with SH have higher incidences of panic attacks, problems with their short-term memory, and concentration problems [9,10]. In the long term, children with SH have higher blood pressure levels and dyslipidemia. Higher levels of triglycerides and LDL can be found, as well as decreased levels of HDL. The mechanism, however, is unknown at this moment [5,11,12,13]. Finally, SH causes higher cardiovascular morbidity and mortality in young adulthood [14]. 

A treatment for SH is lacking, except for the supplementation of thyroxine when TSH rises above 10 mU/L, with some exceptions (e.g., goiter or clear symptoms of hypothyroidism) [15,16]. A meta-analysis in adults with SH studied the benefits of thyroid supplementation and its effect on thyroid related symptoms. In this analysis of 21 studies, the TSH levels decreased to normal ranges after hormone therapy, but the thyroid-related symptoms, such as tiredness, and quality of life did not improve [17]. In a study in children, no relation was shown between the neurocognitive function of children (and their developing brain) and the use of thyroxin therapy [8]. This shows the unclear relationship between clinical complaints and disturbed biochemical thyroid values. 

Alternative approaches to treat SH have been tested, such as the supplementation of nutrients, selenium in particular. Selenium is used for the production of selenoproteins, essential for the protection and metabolism of the thyroid gland. Nutritional sources of selenium are meats, dairy, grains and seafood. Dietary selenium intake measured with food frequency questionnaires showed an inverse association with SH, independent of the intake of energy (kcal) and other nutrients. The more selenium Brazilian adults consumed with their daily food intake, the lower the incidence of SH [18]. When evaluated in a meta-analysis, selenium as a supplement did not alter thyroid function, nor did it improve clinical symptoms [19]. Selenium rich foods (e.g., higher selenium content) instead of a single selenium supplement could therefore be related to prevention of SH. 

Given the incongruence in observed clinical outcomes when comparing nutrient supplementation with foods containing the specified nutrients, such as in the previous examples regarding selenium, we sought to study a broader dietary intervention that included multiple unprocessed and minimally processed foods containing a variety of essential nutrients for optimal thyroid functioning.

The dietary advice consisted of unprocessed food: beef, green vegetables, whole milk and butter. The dietary advice intervention is particularly rich in minerals like selenium, vitamin A, iron and omega-3 fatty acids [20]. We hypothesized that dietary intervention can replenish possible (subclinical) deficiencies and thus improve thyroid functioning. In a previously published non-randomized study we saw favorable results on thyroid functioning with this dietary advice and TSH levels decreased in the intervention group [21]. 

In our outpatient clinic, parents often report tiredness in their child with SH as the main clinical complaint. Therefore, we also looked at tiredness with sub scores on general tiredness, sleep and cognitive functioning. To evaluate the possible negative long-term effects of the dietary intervention, we looked at the BMI and lipid profile to investigate the development of potentially unfavorable dyslipidemia profiles. As far as we know, this is the first randomized controlled trial with a dietary intervention for children with SH. 

## 2. Materials and Methods

### 2.1. Population

The research population consisted of children with SH at the outpatient clinics (general pediatrics) of two medium sized hospitals. Inclusion criteria were age 1–12 years, diagnosis of SH confirmed by a pediatrician, understanding of the Dutch language by the parents. Exclusion criteria were clinical hypothyroidism (FT4 < 10 pmol/L), treatment with thyroid hormone (like (levo)thyroxine), immunological abnormalities, cows’ milk allergy, known or suspected disorder of the intestinal absorption (e.g., celiac disease), disorders requiring a special diet (e.g., metabolic diseases, lactose intolerance) any relevant congenital or anatomical abnormality, chromosomal disorder or severe disease. 

### 2.2. Recruitment and Randomization

General physicians or pediatricians referred the children with SH to the investigators if there was a diagnosis of subclinical hypothyroidism (TSH > 4.2 mU/L and FT4 within the normal range). The children’s parent(s) and/or legal guardian(s) (hereafter named parents) were provided with an information brochure of the study and an appointment with one of the pediatricians participating in the study was scheduled. The pediatrician saw the child in the outpatient clinic, took a history, and performed a physical investigation including measurements of weight and height. If there were any suggestions of an underlying disease causing the subclinical hypothyroidism, the pediatrician investigated and treated the child accordingly. 

The investigator answered any questions parents might have. If parents agreed for the child to participate in the study, informed consent was signed. Parents had a period of one week to rethink the information before signing. After the signing of informed consent, the child was randomized into either the treatment or control group. The children were randomized with the MinimPy minimization software (Minimpy 0.3) on a central computer. The (known and potential) confounders age and BMI were equally distributed between the two groups. 

### 2.3. Study Protocol

After randomization the dietary advice was revealed to the dietary intervention group, but not to the control group. The control group was advised to continue their dietary habits as usual. Parents and subjects of both groups received information about the nature of subclinical hypothyroidism, the natural course, and standard supportive care (conservative management, no intervention required). A diary to record adherence to the dietary advice was handed out to the parents, along with an explanation of how to use it properly. The control group filled out a diary to specify their intake of dairy products, vegetables and meat.

Visits to the clinic were scheduled at inclusion, 3 and 6 months. Laboratory diagnostics were performed at inclusion and about one to two weeks before the visit at 3 months and 6 months. A telephone consultation was scheduled after the inclusion to discuss the lab results; the pediatrician excluded a child from the study if necessary. During each visit, Pediatric Quality of Life (PedQL) multidimensional fatigue scale questionnaires were filled out by the parents, height and weight were measured.

The protocol was approved by the local medical ethics committee and registered in The Netherlands National Trial Register (NL4891 (NTR5138)). All parents gave permission for the study via written informed consent.

### 2.4. Intervention

The intervention was a dietary advice consisting of beef three times a week, green vegetables five times a week, and a daily portion of full fat milk (300 mL) and butter (5 g on each slice of bread). All components in age-appropriate portions according to the national guidelines [22]. All the other dietary habits remained unchanged. Parents were advised to follow it for 6 months. These foods were chosen because they are rich in the most important nutrients for thyroid: iron, selenium, vitamin A, and, to a lesser extent, iodine [20,23,24]. The operating mechanism by which the different food products influence the thyroid or a mechanism of auto-immune thyroiditis are described in Table S1. 

Adherence to the diet was expressed in percentages. These percentages were derived by dividing the number of portions patients provided, according to their diaries, by the total number of portions they were instructed to take. For example, when the children ate green vegetables four times in a specific week, this was 4/5 (80%) of the recommended amount of green vegetables that week. Green vegetables are one of the four products and therefore composed 25% of the total dietary advice, so 80% of 25% = 20% of the total advice. The same calculation was used for the other food groups. The percentages of the four groups were counted, and a total of 100% could be reached per week (4 × 25%). The intake was calculated per week for 6 months, after that the mean was computed. Parents of children in the control group recorded their children’s dietary routine via a grouped checklist for the products advised for the dietary advice group, which was less specific (e.g., what type of milk, butter, meat, or vegetables did they eat on a daily basis). The above described method was used to calculate their mean intake with respect to the parts of the dietary intake they spontaneously took. 

### 2.5. Measurements

Subclinical hypothyroidism is defined as an increased TSH (>4.2 mU/L) and a normal Ft4 (10–25 pmol/L) [1]. Both before the start of the dietary advice, at t = 3 months and t = 6 months, TSH, FT4 and the lipid profile was determined in all children. At t = 0 and t = 6 months, anti-TPO (anti-Thyroid Peroxidase) values were measured. TSH was measured with electro-chemiluminescence sandwich immunoassay and FT4 with 2 step ECLIA (both on COBAS 6000 Roche Diagnostics). Anti-TPO was measured with chemiluminescence immune assay (Advia Centaur, Siemens). The lipids from the lipid profile are total cholesterol, high-density lipoprotein cholesterol (HDL-C), cholesterol/HDL ratio, low-density lipoprotein cholesterol (LDL-C), triglycerides (TG), and non-HDL. The lipid profile was measured by enzymatic colorimetric techniques with the COBAS 6000 (Roche Diagnostics, Almere, The Netherlands). The LDL was calculated with Friedewald’s formula: LDL = total cholesterol − HDL − (0.45 × TG). 

The height of the children was measured with a digital vertical ruler in centimeters to one decimal place. The children were weighed on a digital scale (in kilograms to one decimal place) in underwear and all measurements were performed by a pediatrician. Standard Deviation (SD) scores were used to compare the values with the reference population of Dutch children. The children’s BMI was calculated by dividing their weight in kilograms by the square of their height in meters. The BMI SD-score was calculated on the basis of gender, age, height, and weight. 

### 2.6. Questionnaires

Tiredness questionnaires were filled out by the parents (age-dependent for 2–4 years, 5–7 years, 8–12 years) (recorded by Pediatric QoL Multidimensional Fatigue Scale, kindly provided by Mapi Research Trust). Scores could be noted in five ways: never, almost never, sometimes, almost always, or always. Scores were inversely related; the higher the score on a scale from 0–100, the less tiredness the child showed. 

Parents received a questionnaire with various baseline characteristics (e.g., family history of thyroid disorders, social environment, educational levels of parents, and presence of tiredness). The intervention and control group kept a daily diary about their intake concerning the four components: what kind of milk, butter, meat or vegetables they ate that week.

### 2.7. Statistical Analysis

Data was analyzed with the software program SPSS^®^ for Windows version 25 (IBM, Chicago, IL, USA). Between-group differences in patient characteristics, growth parameters and lipid profiles at baseline were compared with Student’s t-test when normally distributed, or a Mann–Whitney test when not normally distributed. Categorical values were evaluated with crosstabs and Chi-square tests. Repeated measurements of TSH, lipid profile and growth parameters over time were analyzed mixed model repeated measurement analysis. Corrections for BMI-z and TSH1 at t = 0 are used for all PedQL fatigue scale scores, growth parameters (except BMI), thyroid values (except TSH), and lipid profile values during the follow up in time, not at baseline measurements. Correlation between TSH start levels and PedQL total fatigue scores were evaluated with a Pearson correlation test. For all comparisons, a *p*-value ≤ 0.05 was regarded as significant.

Power analysis was performed based on a spontaneous normalization rate of 40% in TSH in children [25]. In a previously performed case-control study with the investigated dietary advice, we found a normalization rate of 72% [21]. An expected difference between the groups at a normalization rate of 35% was used for power analysis. A power of 0.8 was used and with a significance of *p* < 0.05, a minimum of 54 patients was required. 

### 2.8. Outcomes

Primary outcomes were TSH level and FT4 level at t = 0, t = 3 and t = 6 months. Secondary outcomes were the presence or change in anti-TPO (t = 0 and t = 6 months), growth parameters (height, weight, and BMI SD scores), multidimensional fatigue scale questionnaires and the lipid profile at t = 0, t = 3 and t = 6 months.

## 3. Results

### 3.1. Patient Descriptives

In a period of two and a half years (January 2016–September 2018), 65 patients were included. Four patients dropped out of the study, three in the intervention group and one in the control group. One patient thought the study was finished after three months and discontinued the dietary intervention. One patient (the parents) found they already ate healthily and saw no benefits of changing their diet. One patient repeatedly was a no show and the last patient (from the control group) was not motivated to finish the study. Of the included patients, 29 comprised the intervention group and 32 the control group. Patient characteristics did not differ between both groups (Table 1). 

Most children presented with tiredness on our outpatient clinic. All parents reported that their child was tired, but three patients scored close to maximum scores on the PedQL fatigue scale domains, so their tiredness could not be objectified. Their mean scores are mentioned in Table 2.

### 3.2. Outcomes

No significant changes were seen in TSH levels after 6 months between the groups (Table 2). The intervention group started with higher TSH values compared to the control group. In both groups, TSH values decreased over time. No effect was seen by the introduction of the dietary intervention. No significant differences were seen in FT4 values between the groups. Once the TPO antibodies were present, they remained present. No patients became anti-TPO positive during the course of the study or lowered their antibodies. In the control group, one patient increased with his TSH levels above 12, therefore meeting the criteria to start thyroxine supplementation. In the intervention group, all TSH levels stayed below 10. 

### 3.3. Tiredness

During the 6 months of the study, the intervention group showed an improvement over time on all PedQL fatigue scale domains (Table 2). In comparison to the control group, the intervention group showed significant improvement in the total PedQL fatigue scale scores and PedQL sleep scores.

In the control group, most of the PedQL fatigue scale domains and its sub scores remained in the same range during the study period (Table 2). 

The control group started with slightly higher PedQl fatigue scale scores compared to the intervention group, so this group started with higher energy levels compared to the intervention group. To see if there was a relation with the higher PedQL fatigue scale scores and the lower TSH levels, we performed a correlation analysis but there was no correlation (r = 0.0; *p* = 0.99). 

### 3.4. Growth

No significant changes were seen in growth parameters (Table 3). At the start of the study, children in the intervention group had slightly lower heights and weights compared to the control group. This remained the same during the study period. They grew a bit more than expected, in height and weight, but not significantly.

### 3.5. Lipid Profile

As for long term risk factors like disturbed lipid profiles, no significant changes were seen at the end and during the study period. Minor, non-significant trends were seen; total cholesterol values and LDL values increased slightly in the intervention group, but also favorable lipids. HDL increased in time, and the ratio of total cholesterol/HDL as well as triglycerides decreased over time. 

### 3.6. Adherence to the Diet

The overall adherence to the diet was 88.5% in the intervention group and 40% in the control group (*p* < 0.001). The consumed amounts were not normally distributed; some children ate almost 100% of the dietary advice, others only a few components. From the four components of the dietary advice, the control group consumed a median of 58.5% of the beef advice (e.g., 100% beef advice was three times beef per week), 0% whole milk (e.g., 100% = every day 300 mL whole milk), 0% butter (e.g., 100% = every day butter on their bread) and 80% of the green vegetables advice (e.g., 100% = 5 times green vegetables per week). The intervention group consumed a median of 81% of the green vegetables, a median 96.5% of the beef advice, a median 94.2% of the whole milk and a median 94.2% of the butter advice.

## 4. Discussion

While TSH levels of the children with SH decreased during the course of this study, this decrease cannot be attributed to our dietary intervention since both groups decreased in their TSH levels to the same extent. Furthermore, FT4 values did not change during the course of the study. Therefore, advising green vegetables, beef, whole milk and butter did not change thyroid functioning. However, short term clinical parameters such as total PedQL fatigue scale scores and sleep quality, did improve during the dietary intervention, without medication but with a change in dietary habits. In our previously non-randomized study we did see significantly lower TSH levels in the intervention group after adhering to the same dietary advice [21]. Since the study from 2012 was non-randomized, a selection of patients could have occurred. A suggestion for this could be the spontaneous normalization rate in the control group, since that was extremely low compared to the normalization rate of the intervention group. In the present study, we saw similar descending TSH trends in both groups and therefore conclude that the present study represented the findings of other studies much better [25,26].

Hence, the effect of the dietary intervention seemed to work independently of thyroid functioning. The absence of the relation between thyroid hormones and clinical parameters was also described by Feller et al. They reviewed clinical parameters in adults with SH including BMI, blood pressure, general quality of life and/or neurocognitive functioning after supplementation with thyroxine. Even though the biochemical values were normalized, clinical complaints remained unchanged as evaluated in their meta-analysis [17]. Tiredness was investigated in the TRUST study in elderly people with SH. They received low dose thyroxine (25 or 50 ug/day). After 1 year, no improvement was seen in tiredness, yet the mean TSH levels normalized [27]. In our study, tiredness in children was measured in various domains with the PedQL multidimensional fatigue scale: general tiredness, sleep quality, cognitive functioning and the sum of these three (total score). Sleep and the total tiredness score both improved significantly. In tired children without SH, the same dietary advice also improved tiredness scores. In that case-control study, mainly the sleep domain was improved by the diet and the intake of green vegetables and whole milk showed to have the strongest relation with the improvement [28]. In our present study (and in contrast to the aforementioned study), the control group also consumed many green vegetables. This minimal difference in green vegetable intake between the control and intervention group might have suppressed some effects of the dietary intervention, with respect to the findings of the previous study. 

The Mediterranean diet and its effect on fatigue was studied in two studies. Exercise performance (endurance) improved in healthy adults (mean age 28 years), despite similar heart rates and efforts after using a Mediterranean diet for 4 days compared to a Western diet for 4 days [29]. A Mediterranean-style eating pattern also improved fatigue in overweight adults, independent of the intake of red meat (beef or pork) [30]. This implies that our findings are supported by the results of other healthy nutrient-rich dietary habits. A healthy diet can improve fatigue following physical exertion by adding nutrients or eliminating unhealthy products.

Possible mechanisms of nutrition to minimize fatigue are to supplement deficiencies like iron or vitamin D deficiency [31,32]. Antioxidants can be supplied, which reduce free radicals and possibly improve the function of mitochondria [33,34]. Another possible mechanism is by adding n-3 polyunsaturated fatty acids (n-3 PUFA) to prevent metabolic dysfunction of skeletal muscle; however, evidence for this is not convincing [35]. Our studied dietary advice is rich in minerals and vitamins like iron and vitamin D, has a favorable n-3 PUFA n-6 PUFA ratio and contains antioxidants [20].

In the past, moderately elevated levels of TSH (in subclinical hypothyroidism) have been found to be a consequence rather than a cause of weight gain [26]. In our study, we corrected TSH levels for BMI measures. This did not influence the final results. This was underscored by higher TSH levels in our intervention group, while BMI levels of the intervention group were lower compared to the control group. 

Cardiovascular risks are a possible consequence of SH in children, although consensus has not been reached yet [11,12,36]. Besides the thyroid functioning, an elevated BMI is a possible additional risk factor. Our dietary advice consisted both of high energy products (whole milk and butter) and low energy products (green vegetables and beef). When all four components are consumed, it should not largely increase the total energy intake. As a possible result, the intervention group began with and maintained their BMI a little above the 0 SD line (normal values). BMI was therefore not influenced by the dietary advice. Dyslipidemia is another possible risk factor, especially in the presence of low HDL and/or high cholesterol/HDL ratios. In the children following our dietary intervention, HDL tended to increase, and the cholesterol/HDL ratio tended to decrease, both moving towards prognostically favorable values. Although these changes were not significant, they indicate a favorable effect that is in line with previous findings [28].

### 4.1. Limitations and Future Directions

This study included several possible sources of bias. First, the design of the study is open and not blinded. Since the parents had to prepare the food based on the dietary advice, it was not possible to blind the studied intervention. Secondly, the children in the control group had higher PedQL multidimensional fatigue scale scores at the start of the study compared to the intervention group. The parents filled out the PedQL questionnaire after they were randomized, and we noticed these differences after processing the questionnaires. Thirdly, we noted some overlap in diet: both the control group and the intervention group ate considerable amounts of green vegetables and beef. The big differences were found in the consumed amount of whole milk and butter and, to a lesser extent, beef. The control group was not informed about the diet, but they might have heard about it informally. At last, patients were studied for 2 years. The seasonal influence of patient enrolment or follow up was therefore minimized, since both groups were included and randomized for these 2 years. Possible seasonable influences were therefore divided over both groups. 

A suggestion for further research could be to investigate other risk markers for cardiovascular disease in SH-like serum concentrations of dimethylarginine (a marker for endothelial dysfunction) or intima thickness. These measurements are influenced by prolonged elevated TSH levels. What will happen to these markers when TSH stays high? TSH levels are not influenced by our dietary advice, but what about these risk markers? 

### 4.2. Implications

As far as we know, this is the first study introducing a lifestyle (dietary) intervention as a possible approach to alleviate complaints related to SH. Up until now, the only treatment for children with SH is to keep track of thyroid hormonal levels and to provide thyroxine medication when TSH increases above 10 IU/L. However, for the ‘in-between’ children with levels above 4.2 but below 10 IU/L, generally no medication is given, and parents cannot do anything to alleviate the complaints of their children. For these children, the dietary intervention tested in this study can help to reduce tiredness, and hence improve wellbeing, without increasing the risk factors of long-term consequences. 

## 5. Conclusions

In children with SH, a dietary intervention consisting of green vegetables, beef, whole milk and butter did not improve thyroid function in terms of thyroid hormone production, but it did reduce tiredness, as measured with the PedQL fatigue scores. Hence, this lifestyle (dietary) intervention could be a possible tool to improve the wellbeing of children with SH, independent of thyroid function.

## Figures and Tables

**Table 1:** Patient characteristics (chi square tests; sex, two-parent family, parental education, tiredness, family history, presence anti-TPO. Independent *t*-test; age. Mann–Whitney test; Total Cholesterol (TC), high-density lipoprotein (HDL), cholesterol/HDL ratio, triglycerides (TG), low-density lipoprotein (LDL), height, weight and BMI).

	Intervention Group	Control Group	*p*-Value
	*n* = 29	*n* = 32	
Male:female	15:14	16:16	0.89
Age in years (SD)	7.7 (3.1)	8.1 (3.3)	0.67
2-Parents family	27 (51%)	26 (49%)	0.23
Parental education (only primary or high school)	6 (20.6%)	1 (3.3%)	0.32
Tiredness (according to parents)	23 (79%)	22 (73%)	0.59
Positive family history for thyroid diseases (%)	16 (55%)	15 (52%)	0.55
TSH start (mIU/L) (SD)	6.14 (1.4)	5.69 (1.4)	0.15
FT4 start (pmol/L) (SD)	16.6 (2.1)	15.7 (1.6)	0.06
Anti-TPO positive (%) start	2 (7%)	4 (12%)	0.46
Total Cholesterol (mmol/L) (median, IQR)	4.2 (3.7–4.6)	4.3 (3.8–4.9)	0.28
HDL-C (mmol/L) (median, IQR)	1.4 (1.2–1.8)	1.4 (1.2–1.8)	0.80
Cholesterol/HDL ratio (mmol/L) (median, IQR)	3.0 (2.3–3.5)	3.1 (2.4–4.0)	0.61
TG (mmol/L) (median, IQR)	0.9 (0.7–1.3)	1.0 (0.6–1.4)	0.88
LDL-C (mmol/L) (median, IQR)	2.2 (1.8–2.4)	2.4 (1.7–3.0)	0.37
Height (cm) (median, IQR)	127 (113–139)	132 (115–148)	0.25
Weigth (kg) (median, IQR)	24.9 (21–34)	31.1 (20–46)	0.16
BMI (kg/m^2^) (median, IQR)	16.0 (15–17)	17.0 (16–20)	0.05

**Table 2:** Thyroid hormones and fatigue scale (with 95% Confidence Interval) scores during the 6-month intervention (mixed model analysis). *p*-values express the differences between both groups.

	Intervention Group			Control Group			*p*-Value
	t = 0	t = 3	t = 6	t = 0	t = 3	t = 6	
TSH (mIU/L)	6.1 (5.6–6.6)	5.5 (4.8–6.1)	5.2 (4.5–6.0)	5.7 (5.2–6.2)	5.0 (4.3–5.6)	4.8 (4.1–5.5)	0.98
FT4 (pmol/L)	16.6 (16–17)	17.1 (16–18)	16.6 (16–17)	15.7 (15–16)	16.0 (15–17)	15.4 (15–16)	0.51
PedQL total	61.8 (55–68)	67.9 (61–75)	74.1 (67–81)	70.9 (65–77)	70.5 (64–77)	72.8 (66–80)	0.04
PedQl general	59.4 (51–67)	65.6 (57–74)	72.9 (65–81)	67.2 (60–75)	67.9 (60–76)	72.4 (65–80)	0.14
PedQl sleep	62.5 (55–69)	69.9 (63–77)	77.8 (71–84)	72.5 (66–79)	73.1 (66–79)	75.9 (70–82)	0.03
PedQl cognitive	62.9 (53–73)	67.1 (57–76)	70.4 (60–81)	73.9 (65–83)	71.4 (62–80)	71.1 (61–81)	0.19

**Table 3:** Growth parameters and lipid profile (both with 95% CI) during the 6-month intervention (mixed model analysis). *p*-values express the differences between both groups.

	Intervention Group			Control Group			*p*-Value
	t = 0	t = 3	t = 6	t = 0	t = 3	t = 6	
SD height	−0.28 (−0.6–0.1)	−0.29 (−0.6–0.1)	−0.21 (−0.6–0.2)	0.1 (−0.2–0.2)	0.16 (−0.2–0.5)	0.11 (−0.2–0.5)	0.18
SD weight	0.18 (−0.3–0.7)	0.2 (0.3–0.7)	0.28 (−0.3–0.8)	0.78 (0.3–1.2)	0.83 (0.4–1.3)	0.8 (0.3–1.3)	0.24
SD BMI	0.34 (−0.1–0.8)	0.37 (−0.1–0.9)	0.4 (−0.1–0.9)	0.83 (0.4–1.3)	0.84 (0.4–1.3)	0.84 (0.3–1.3)	0.89
TC (mmol/L)	4.23 (4.0–4.5)	4.33 (4.1–4.6)	4.34 (4.1–4.6)	4.29 (4.1–4.5)	4.19 (4.0–4.4)	4.22 (4.0–4.4)	0.16
HDL (mmol/L)	1.4 (1.3–1.6)	1.44 (1.3–1.6)	1.52 (1.4–1.7)	1.47 (1.3–1.6)	1.43 (1.3–1.6)	1.46 (1.3–1.6)	0.20
cholesterol/HDL ratio	3.21 (2.8–3.6)	3.15 (2.8–3.5)	2.99 (2.7–3.3)	3.19 (2.8–3.5)	3.14 (2.8–3.4)	3.09 (2.8–3.3)	0.78
TG (mmol/L)	1.14 (0.8–1.4)	1.32 (0.9–1.3)	0.91 (0.7–1.1)	1.15 (0.9–1.4)	0.98 (0.8–1.2)	0.98 (0.8–1.2)	0.35
LDL (mmol/L)	2.31 (2.1–2.5)	2.38 (2.–2.6)	2.42 (2.2–2.6)	2.3 (2.1–2.5)	2.31 (2.1–2.5)	2.3 (2.1–2.5)	0.57

## Supplementary Materials

The following are available online at https://www.mdpi.com/1660-4601/17/10/3689/s1, Table S1: Operating mechanisms of food products on the thyroid or immune system.
