# Supplementary material for: A Lifestyle (Dietary) Intervention Reduces Tiredness in Children with Subclinical Hypothyroidism, a Randomized Controlled Trial
Source: Int J Environ Res Public Health. 2020 May 23;17(10):3689. doi: 10.3390/ijerph17103689 (PMC7277354; doi:10.3390/ijerph17103689)
Supplement: Supplementary file 1 [file ijerph-17-03689-s001.pdf]

Table S1. Operating mechanisms of food products on the thyroid or immune system.

Products/nutrition	Suggested mechanism of action and association with thyroid function.
**Green vegetables**	*5x/week for supper*
**Vitamin A**	Improves efficacy of iodine uptake and can have a beneficial impact on thyroid function and size[1-3]. Modulates TSH production by influencing the expression of pituitary TSHβmRNA[4].
**Vitamin C**	Improves iron-uptake, in persons with hypothyroidism, it improves the abnormalities in serum free T4, T3 and TSH concentrations[5].
**Zinc**	Zinc levels are positively correlated with free T3-levels[6].
**Beef**	*3x/week for supper*
**Iron**	Providing iron along with iodine results in greater improvements in thyroid function[3]. Both iron deficiency and iron excess can influence the functioning of the innate and adaptive arms of the immune system, thereby it contributes to a more stable thyroid function[7, 8].
**Iodine**	Chronic iodine deficiency increases the TSH concentration [9].
**Vitamin A, C, zinc**	Stated above.
**Selenium**	Selenium deficiency impairs thyroid hormone metabolism by inhibiting the synthesis and activity of the iodothyroinine deiodinases, which convert T4 into T3 [10].
**Whole dairy products**	*200 mL whole milk a day / whole butter on bread daily*
**Vitamin D**	Has a role in the maintenance of immune-homeostasis. Deficiency of vitamin D is linked to the presence of antithyroid antibodies and abnormal thyroid functions [11].
**Fatty acids**	Signs of faster immune maturation with supplementation[12].

References

1. Zimmermann, M. B., Interactions of vitamin A and iodine deficiencies: effects on the pituitary-thyroid axis. *Int J Vitam Nutr Res* **2007,** *77* (3), 236-40.
2. Zimmermann, M. B.; Wegmuller, R.; Zeder, C.; Chaouki, N.; Torresani, T., The effects of vitamin A deficiency and vitamin A supplementation on thyroid function in goitrous children. *J Clin Endocrinol Metab* **2004,** *89* (11), 5441-7.
3. Hess, S. Y., The impact of common micronutrient deficiencies on iodine and thyroid metabolism: the evidence from human studies. *Best Pract Res Clin Endocrinol Metab* **2010,** *24* (1), 117-32.
4. Brown, N. S.; Smart, A.; Sharma, V.; Brinkmeier, M. L.; Greenlee, L.; Camper, S. A.; Jensen, D. R.; Eckel, R. H.; Krezel, W.; Chambon, P.; Haugen, B. R., Thyroid hormone resistance and increased metabolic rate in the RXR-gamma-deficient mouse. *J Clin Invest* **2000,** *106* (1), 73-9.
5. Jubiz, W.; Ramirez, M., Effect of vitamin C on the absorption of levothyroxine in patients with hypothyroidism and gastritis. *J Clin Endocrinol Metab* **2014,** *99* (6), E1031-4.

6. Ertek, S.; Cicero, A. F.; Caglar, O.; Erdogan, G., Relationship between serum zinc levels, thyroid hormones and thyroid volume following successful iodine supplementation. *Hormones (Athens)* **2010,** *9* (3), 263-8.

7. Ekiz, C.; Agaoglu, L.; Karakas, Z.; Gurel, N.; Yalcin, I., The effect of iron deficiency anemia on the function of the immune system. *Hematol J* **2005,** *5* (7), 579-83.

8. Cherayil, B. J., Iron and immunity: immunological consequences of iron deficiency and overload. *Arch Immunol Ther Exp (Warsz)* **2010,** *58* (6), 407-15.

9. Zimmermann, M. B., Iodine deficiency. *Endocr Rev* **2009,** *30* (4), 376-408.

10. Arthur, J. R.; Nicol, F.; Beckett, G. J., The role of selenium in thyroid hormone metabolism and effects of selenium deficiency on thyroid hormone and iodine metabolism. *Biol Trace Elem Res* **1992,** *34* (3), 321-5.

11. Yin, K.; Agrawal, D. K., Vitamin D and inflammatory diseases. *J Inflamm Res* **2014,** *7*, 69-87.

12. Damsgaard, C. T.; Lauritzen, L.; Kjaer, T. M.; Holm, P. M.; Fruekilde, M. B.; Michaelsen, K. F.; Frokiaer, H., Fish oil supplementation modulates immune function in healthy infants. *J Nutr* **2007,** *137* (4), 1031-6.
